# Persistent homology as a new method of the assessment of heart rate variability

**DOI:** 10.1371/journal.pone.0253851

**Published:** 2021-07-22

**Authors:** Grzegorz Graff, Beata Graff, Paweł Pilarczyk, Grzegorz Jabłoński, Dariusz Gąsecki, Krzysztof Narkiewicz

**Affiliations:** 1 Faculty of Applied Physics and Mathematics & BioTechMed Center, Gdańsk University of Technology, Gdańsk, Poland; 2 Department of Hypertension and Diabetology, Medical University of Gdańsk, Gdańsk, Poland; 3 Faculty of Applied Physics and Mathematics & Digital Technologies Center, Gdańsk University of Technology, Gdańsk, Poland; 4 Insitute of Science and Technology, Klosterneuburg, Austria; 5 Department of Neurology for Adults, Medical University of Gdańsk, Gdańsk, Poland; Utrecht University, NETHERLANDS

## Abstract

Heart rate variability (hrv) is a physiological phenomenon of the variation in the length of the time interval between consecutive heartbeats. In many cases it could be an indicator of the development of pathological states. The classical approach to the analysis of hrv includes time domain methods and frequency domain methods. However, attempts are still being made to define new and more effective hrv assessment tools. Persistent homology is a novel data analysis tool developed in the recent decades that is rooted at algebraic topology. The Topological Data Analysis (TDA) approach focuses on examining the shape of the data in terms of connectedness and holes, and has recently proved to be very effective in various fields of research. In this paper we propose the use of persistent homology to the hrv analysis. We recall selected topological descriptors used in the literature and we introduce some new topological descriptors that reflect the specificity of hrv, and we discuss their relation to the standard hrv measures. In particular, we show that this novel approach provides a collection of indices that might be at least as useful as the classical parameters in differentiating between series of beat-to-beat intervals (RR-intervals) in healthy subjects and patients suffering from a stroke episode.

## 1 Introduction

Computational Topology [1] is a modern branch of science that combines the century-long experience in Algebraic Topology with the new capabilities of contemporary computers. It provides novel methods for extracting succinct yet meaningful information from complex data. Persistent Homology (see e.g. [2]) is the main concept that allows for multiscale data analysis, and a fundamental mathematical tool of Topological Data Analysis (TDA). This powerful tool encompasses the concept of topological persistence for sublevel sets of real-valued functions, developed already in the 1990s [3–5] and considered a fundamental idea that was further generalized into Persistent Homology. This idea is often used in tutorials and introductions to Persistent Homology; see e.g. [6] or [7, Section 5.2]. The topological persistence is especially well-suited for direct time series analysis; see e.g. [8–10] and references therein.

Persistent homology is an extension of classical homology that describes topological space by counting connected components, holes, tunnels, voids, and their higher dimensional counterparts [1]. For example, if one considers the existing methods of hrv analysis, the persistent homology approach in the two-dimensional case may be interpreted as the statistics of the number of holes is the Poincaré diagram relative to the resolution of the data.

In this paper, we limit our attention to homology in dimension 0. One of the main reasons for our choice is the fact that the 0-dimensional homology has natural physiological interpretation in terms of heart rate variability, unlike the higher-dimensional counterparts; see Section 4 for the discussion of this. Moreover, the 0-dimensional approach is already highly non-trivial and provides considerable amount of information, as it was shown in some studies on biomedical data. For example, 0-dimensional homology method was used to support the obesity treatment [11], analyze brain artery trees [12], or differentiate young patients in research on the attention-deficit/hyperactivity disorder (ADHD) [13].

While 0- and 1-dimensional analysis is frequently used in signal processing, the application of higher-dimensional approach is challenging. On the one hand, some important relations in data might be found that are not revealed in lower dimensions (see, for example, [14]); on the other hand, in higher dimensions the analysis may be much more difficult from the computational point of view [15]. Moreover, little is known about the interpretation of higher-dimensional Betti numbers apart from conjecturing some kind of recurrent behavior [16], such as periodicity [15] or quasi-periodicity [17]. Another problem is finding appropriate embedding dimension in which the analysis is meaningful; for example, in the case study of ECG data conducted in [18] it was found out that the results strongly depended on the choice of the embedding dimension (the dimensions considered there ranged from 3 to 7).

The 0-dimensional homology considered in the paper describes connected components of sublevel sets of the graph of a continuous real-valued function of one variable. This function is the piece-wise linear interpolation of the mapping that assigns consecutive RR intervals to the time points at which they appear. When the sublevel set grows, new connected components are created or existing components merge (and then one of them disappears). We assign two thresholds to each connected component: one that represents its birth (creation) time, and the other one that represents its death (destruction) time. The persistence diagram consists of all the (birth, death) pairs, in our considerations also interpreted as intervals; see Section 2.1 for details.

The study of hrv has a long tradition with the use of various linear and nonlinear methods [19]. On the other hand, the repertoire of methods used to complement the existing ones is still growing [20].

In this paper we study the usefulness of persistent homology as a novel tool that has already been successfully applied in many branches of science.

Although nonlinear and topological methods have already been used in data processing for several years [21], the application of persistent homology to hrv data is a matter of recent years only. For example, in [22, 23], persistent homology was applied to solve the problem of automatic sleep stage classification. The method was used also to study some pathological states. It turned out to be a valuable method for identifying atrial fibrillation [24], and enabled personalized electrocardiographic signal classification toward arrhythmia detection [25]. In [26] the persistent homology of networks formed by ordinal partitions for ECG were studied, and used to detect difference between epileptic and healthy patients.

The character of our work is primarily methodological, with the aim of initiating the systematic study of the persistent homology as a tool that could be useful in hrv analysis.

In our previous paper [27], published in the proceedings of ESGCO, we summarized a preliminary study that indicated for the first time the potential usefulness of the topological methods in the analysis of ECG data. However, in that study we only used 4 most generic topological parameters, and did not conduct systematic study of their interpretation in the context of hrv analysis. In this study we expand the collection of the parameters substantially, and describe many topological indices that characterize persistent homology diagrams obtained from RR series. We do not only include indices available in the literature, but also define new ones that reflect the specificity of the RR time series data. These are, in particular, the family of triangular indices and the signal to noise ratio. In fact, there are many possible indices that could be defined within the TDA framework. Our aim is to indicate a collection of natural quantities that is small enough to be handled efficiently, and also somehow reflects the geometry of RR series.

In order to investigate the most important features of the topological methods, we consider short-time ECG recordings in healthy volunteers and patients with the ischemic stroke episode, the latter presenting a large variety of heart rate variability alterations. Acute ischemic stroke is a leading cause of serious disability and the major cause of mortality in adults. Autonomic imbalance represented by decreased heart rate variability has been reported in stroke patients. The hrv parameters, assessed from continuous short-term recordings in the acute phase of ischemic stroke, can also differentiate groups with various neurological outcomes [28]. However, most of standard HRV parameters are sensitive to the mode of signal collection and edition and, at many times, the interpretation of the results must include the simultaneous analysis of respiratory rate. In order to draw conclusions about the applicability of topological methods to hrv, we first investigate the relation of topological and standard hrv indices, and next we verify how methods based on persistent homology manage to distinguish between the studied groups. Previous studies have shown the association of ischemic stroke (brain ischemia) with an impairment of cardiac autonomic balance showed by reduced heart rate variability and impaired baroreceptor sensitivity. Among others, our team have demonstrated that both standard and non-linear HRV parameters might have prognostic value as it comes to short- and long-term functional outcome in post-stroke patients [28]. However, there is still need for future development of good non-invasive predictors of stroke and its complications. We believe that novel methods of HRV assessment might provide additional insight into post-stroke heart rate changes not only related to the change of cardiovascular system, but also from different origins, including respiratory or humoral ones. Indeed, application of topological methods to the analysis of biological, medical, or physical models may provide new insights and valuable results that might compete with the results of analysis conducted by classical methods; see [29–31] for some examples. As a consequence, in order to claim our research successful, we aim at providing experimental evidence that the results obtained using the topological parameters are at least as good as the results obtained using the standard hrv parameters. We consider this one of the essential steps for further development of clinically useful models for prediction and detection of stroke and its complications.

The paper is organized as follows. In the first section we provide precise definition of the persistent homology method and introduce the parameters based on persistence diagram in details. Then we describe the data set that is used to benchmark the topological method, and we also list step by step all the operations that we apply to the data. The second section is devoted to the comparison of topological indices and standard hrv measures tested on groups of healthy subjects and patients with the episode of ischemic stroke. The obtained results are discussed and concluded in the last sections.

## 2 Materials and methods

### 2.1 Persistent homology

**Fig 1:**
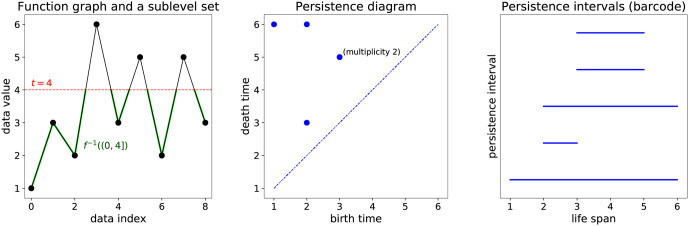
Persistent homology example. (a) Time series data (*r*_0_, …, *r*_8_), the continuous function *f* obtained by linear interpolation, and the sublevel set *f*^−1^((0, 4]); note the four connected components that are ‘alive’ at this time. (b) The corresponding persistence diagram containing five birth-death points; note the repetition of (3,5): both occurrences of this point correspond to connected components that are born, respectively, at indices 4 and 8. (c) The persistence intervals for all the birth-death pairs.

Let f:[a,b]→R be a real-valued function defined on a compact interval. This includes the case of a time series (*r*_0_, …, *r*_*n*_), which can be extended to a continuous function f:[0,n]→R by linear interpolation between the data points. For each t∈R, define the corresponding *sublevel set* as *f*^−1^((−∞, *t*]). This is the part of the graph of *f* where the values of *f* do not exceed *t*. See Fig 1(a) for an example. Note that whenever *t*_1_ ≤ *t*_2_, there is the inclusion *f*^−1^((−∞, *t*_1_]) ⊆ *f*^−1^((−∞, *t*_2_]).

Topological persistence (introduced in [3–5]) focuses on tracking the changes in topology throughout the family of all the sublevel sets of *f*. Let us briefly review this concept. When *t* increases, new connected components can be born (due to crossing local minima of *f*), or existing connected components can join each other (due to crossing local maxima of *f*); in the latter case we say that the component that was born later dies, and if both were born at the same time, we arbitrarily choose one of them. A succinct representation of this process is a list of pairs: a birth time of each connected component paired with its death time. Observe that if *t* < min *f*([*a*, *b*]) then *f*^−1^((−∞, *t*]) = ∅. Moreover, if *t* ≥ max *f*([*a*, *b*]) then *f*^−1^((−∞, *t*]) comprises of the entire graph of *f* and thus has precisely one connected component; we pair the birth time of this component with the maximum value of *f*.

The birth-death times of connected components can be depicted as points in the birth-death plane, called *persistence diagram*, as shown in Fig 1(b). Note that some points may have multiplicity greater than 1 if they correspond to different connected components with the same birth and death times. Points close to the diagonal come from connected components with short lifetimes, and these correspond to small fluctuations in the value of *f*; these features that can only be seen at fine scales, and thus might sometimes be considered noise in the data. Points farther apart from the diagonal come from connected components with long lifetimes and thus correspond to large-scale features in the data. It is also convenient to consider the collection of birth-death intervals (with repetitions), called *persistence intervals* or *barcode*, as illustrated in Fig 1(c). We use the Dionysus 2 software by Dmitriy Morozov to compute the persistence intervals, see [32, 33].

We remark that instead of considering connected components of sublevel sets, one might directly pair the local minima and maxima of *f*, although such an approach seems more technical; see e.g. [11].

### 2.2 Persistent homology indices

In this section we introduce some quantities that characterize persistence diagrams. Later we will study their relation to some classical indices that describe heart rate variability.

Let (*r*_0_, …, *r*_*n*_) be a sequence of RRs, and let ((*b*_1_, *d*_1_), …, (*b*_*k*_, *d*_*k*_)) be the computed persistence intervals, where *b*_*i*_ are the birth times, and *d*_*i*_ are the death times of the connected components of the sublevel sets, as explained in Section 2.1. For simplicity of further definitions, let us assume that the sequence of persistence intervals is sorted by the lengths of the persistence intervals, denoted by *l*_*i*_ ≔ *d*_*i*_ − *b*_*i*_ for *i* = 1, …, *k*. That is, we have *l*_*i*_ ≤ *l*_*j*_ for *i* ≤ *j*.

Let us point out the fact that several topological features can be computed from the sequence of persistence intervals, but a good choice of particular quantities depends on a specific application; see e.g. [34, 35]. We use both the previously defined topological indices, cf. [36], and some new ones as well, introduced in this study to characterize the RR data; see Sections 2.2.5 and 2.2.8 for the latter.

The first and most obvious index is the *number of intervals*, the number *k* in the formulas above. In the subsequent sections we define several more advanced indices.

#### 2.2.1 Length parameters: *Longest interval, length mean, length median, length stdev, length sum*

One of the most frequently used parameters is the length of the longest persistent interval *l*_*k*_ = *d*_*k*_ − *b*_*k*_ (denoted as *longest interval* for short). Observe that *l*_*k*_ equals max *r*_*i*_−min *r*_*i*_, which indicates the size of the range of the sequence ${\left(r_{i}\right)}_{i=0}^{n}$. This parameter is a well-known indicator in hrv analysis, reflecting respiratory sinus arrhythmia. Usually denoted as “HR Max−HR Min,” it stands for the difference between the highest and lowest lengths of RR intervals [37]. The length of the longest persistent interval may sometimes be used as a normalizing factor.

The mean (*length mean*), median (*length median*) and the standard deviation (*length stdev*) of the lengths of persistence intervals {*l*_1_, …, *l*_*k*_} are other length-type parameters. These quantities indicate typical lengths of both short-term and long-term increases and decreases found in the RR sequence, as well as the variability in size of increases and decreases in this sequence.

We also compute the sum of lengths of all the persistence intervals (*length sum*), which is known in the literature as the 1st persistence moment, or degree-1 total persistence of the function (see [38, pg. 133]). Since this sum grows with the RR sample length, normally one would have do divide this sum by the number of RR intervals (or apply a similar scaling factor), and we would thus call it *length sum per rr*. However, since in our case the number of considered RRs is essentially the same for each patient, we do not need to make this correction.

#### 2.2.2 Proportion between the longest intervals: *2/1 ratio, 3/1 ratio*

The proportion between the length of the second longest persistence interval to the length of the longest one: *l*_*k*−1_/*l*_*k*_, indicates how much of the global minimum–maximum span can be achieved using another minimum or another maximum (or both). We denote it as *2/1 ratio*.

Let us point out the fact that some topological indices are sensitive to the existence of outliers, the very high values of episodic RR intervals. This obviously applies to the longest interval. That is why the *2/1 ratio* parameter is introduced. Its role is to provide information on the increase in length achieved in two maximal values.

In some applications, analogous quantity is considered regarding the proportion between the 3rd longest and the 1st longest persistence intervals (we denote it as *3/1 ratio*). Considering the 2nd and 3rd longest intervals in addition to the longest one is not an unusual practice; see, for example [34, Table 1].

**Table 1:** Correlations between selected topological and standard hrv parameters in healthy individuals.

	Topological and standard HRV parameters in healthy individuals (CON group)-correlation coefficients R (ns: non-significant correlation; strong and very strong correlations written in bold)							
	mean nni	sdnn	pnni 50	rmssd	lfnu	hfnu	lf hf ratio	total power
number of intervals	0,37	0,02	ns	ns	-0,31	0,31	-0,31	ns
length median	0,41	**0,68**	**0,91**	**0,92**	-0,50	0,50	-0,50	**0,74**
length stdev	0,31	**0,88**	**0,80**	**0,80**	ns	ns	ns	**0,98**
length sum	0,50	**0,72**	**0,98**	**0,98**	-0,46	0,46	-0,46	**0,79**
frac5%	ns	**0,68**	**0,84**	**0,82**	-0,37	0,37	-0,37	**0,80**
triangle width	ns	0,59	ns	ns	ns	ns	ns	ns
triangle height	0,32	**0,74**	**0,86**	**0,86**	ns	ns	ns	**0,92**
triangle location	**0,98**	0,32	0,47	0,50	ns	ns	ns	ns
triangle misalignment	0,40	**0,95**	**0,74**	**0,74**	ns	ns	ns	**0,90**
signal to noise	ns	**0,74**	**0,77**	**0,75**	ns	ns	ns	**0,88**

#### 2.2.3 Persistent entropy, normed persistent entropy: *Pers entropy, normed entropy*

Entropy is one of the most common measures of irregularity or complexity. Many of its versions were successfully applied in the hrv analysis [39–42]. In our approach, we use topological persistent entropy (see e.g. [43, Definition 3]), which we call *pers entropy* for short. It is calculated according to the formula

$$\begin{array}{c} h\left(r\right)=\sum_{i=1}^{n}-\frac{l_{i}}{L}\log_{2}\frac{l_{i}}{L}, \end{array}$$

where *l*_*i*_ is the length of the *i*-th persistence interval, and *L* is the sum of the lengths of all the persistence intervals. It is an adaptation of classical Shannon entropy in computer science (or Shannon index in ecology) to the context of persistent homology. It is a measure of complexity of the set of lengths of persistence intervals. The higher the value, the more random-like the distribution of these lengths appears. We would like to remark that the persistent entropy is different from *sample entropy* and *approximate entropy* of a time series (see e.g. [40]), and is not directly related to the complexity of the series of RRs.

We also compute a normed version of the persistent entropy, which we call *normed entropy*. It is the persistent entropy divided by log_2_(*L*).

#### 2.2.4 Relative numbers of long intervals: *Frac5%, frac100, frac200*

We set up a threshold that distinguishes very short intervals from those of considerable length; *length threshold* is the 5% of the length of the longest interval. Then we take the index *p* such that *l*_*i*_ ≤ 0.05 *l*_*k*_ for *i* < *p*, and *l*_*i*_ > 0.05 *l*_*k*_ for *i* ≥ *p*. Limiting one’s attention to intervals whose persistence exceeds some nonnegative threshold is a common practice, and is used, for example, in the definition of the *p*-th persistence moment in [38, pg. 133].

We define *frac5%* as the number of intervals longer than 5% of the longest one, relative to the number of all the intervals: (*k* − *p* + 1)/*k*. This quotient corresponds to the number of considerable increases or decreases in RRs in comparison to all the increases and decreases, including both long-term changes and small fluctuations. We also use the length threshold in the computation of some other parameters in order to limit our attention to large-scale fluctuations in RRs as opposed to “noise”; see Sections 2.2.5–2.2.7.

At this point we remark that changing the threshold of 5% to another value, for example, 10%, results in a change in the individual values of the computed parameters; this change is in some cases considerable. This should be in fact expected, because there is no reason for the distribution of interval lengths to be uniform. A numerical test shows that two features are especially affected: *frac5%* and *signal to noise*, the latter introduced in Section 2.2.5. However, the results of machine learning described in Section 2.3 and illustrated in Figs 6–9 change very little. This is good news, because it suggests that the important features are indeed captured correctly independent of the actual threshold value applied.

Similarly, we define *frac100* as the fraction of intervals whose length is at least 100 ms, and *frac200* as the fraction of intervals whose length is at least 200 ms. The advantage of these indices is the independence of the global scale of variability of RRs. Using absolute values of differences in designing hrv measures is a common practice; for example, the percentage of successive RR intervals that differ by more than 50ms, denoted in the literature as pnni 50, is one of typical hrv time-domain measures; see e.g. [19].

#### 2.2.5 Signal to noise ratio (*signal to noise*)

Small persistence intervals correspond to small fluctuations in the sequence of RRs, and therefore may be regarded as “noise”. We introduce a quantity that measures the amount of changes in RRs encompassed by long intervals in comparison to the cumulative changes in RRs resulting from these small fluctuations. For that purpose, we compute the total length of all the intervals longer than the threshold defined above (that is, 5% of the length of the longest interval) relative to the total length of all the intervals whose length does not exceed the threshold:

$$\begin{array}{c} \left(\sum_{i=p}^{k}l_{i})/(\sum_{i=0}^{p-1}l_{i}\right) \end{array}$$

This definition is inspired by the notion of signal-to-noise ratio that compares the level of the desired signal to the level of the undesired noise affecting the signal, and is well known in engineering. The “power” of a persistence interval is measured as its length in our approach. Although its interpretation is different in our case (as discussed in Section 4), this analogy justifies the name *signal to noise* that we use for this quotient.

#### 2.2.6 Middle point indices: *Middle mean, middle stdev*

Middle point indices are defined as the mean and the standard deviation of the middle point values of all the persistence intervals that are longer than 5% of the longest interval: ${\left(\left(d_{i}+b_{i}\right)/2\right)}_{i=p}^{k}$. This sequence consists of central values around which considerable increases or decreases occur in the RR sequence, and the mean and the standard deviation characterize their location and dispersion.

#### 2.2.7 Birth-death pair indices: *Birth mean, birth stdev, death mean, death stdev*

We consider the mean and the standard deviation of the birth times and of the death times in the birth-death pairs, corresponding to the left-hand-side and the right-hand-side endpoints of the persistence intervals longer than 5% of the longest interval: ${\left((b_{i},d_{i})\right)}_{i=p}^{k}$. These quantities characterize the locations and dispersion at those local minima and local maxima, respectively, that are not part of small fluctuations in the RR sequence. These indices are among the simplest features suggested to be extracted from persistence barcodes; see e.g. [36, Section 4.1.1].

#### 2.2.8 Topological triangle indices: *Triangle width, triangle height, triangle location, triangle proportion, triangle misalignment*

**Fig 2:**
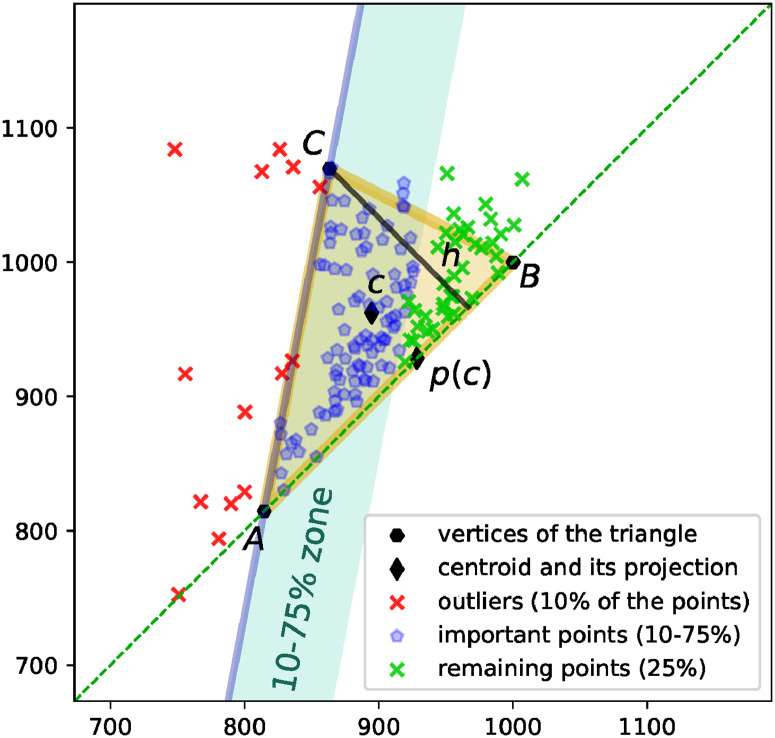
The topological triangle *T*(*D*) that reflects the shape of the set of points *D* in the persistence diagram. See explanations in text.

Classical methods commonly used in hrv analysis include some geometric methods. This is in particular the *triangular index* (a triangular interpolation of the RR interval histogram) and the *Poincaré plot* [19]. Geometric patterns that are revealed during such analysis turn out to carry important information about the structure of the heart rhythm. We follow this line of research and introduce a new geometric index for persistent diagrams that we call the *topological triangle* and denote as *T*(*D*). It provides a simple yet meaningful visual description of the location and shape of the set of points *D* in the persistence diagram. The idea is that the triangle *T*(*D*) should enclose the set of points *D* as tight as possible, with a few points left outside. Indeed, the set *D* often seems to form a filled triangle, which justifies the choice of the shape of the triangle in our approach, as opposed to an ellipse or a rectangle. The procedure for the construction of the topological triangle is as follows; see Fig 2.

Given an angle *φ* ∈ [−*π*/2, 0), consider a line *k* that intersects the diagonal line *d* ≔ {*x* = *y*} at the angle *φ* and that 10% of the points in the persistence diagram *D* are located at the left-hand side of the line, and 90%—at the right-hand side. See line *AC* in Fig 2, with the 10% points labeled as outliers. Symmetrically, given an angle *ψ* ∈ (0, *π*/2], consider a line *l* that intersects the diagonal line *d* ≔ {*x* = *y*} at the angle *ψ* and that 10% of the points in the persistence diagram *D* are located at the right-hand side of the line, and 90%—at the left-hand side (see line *BC* in Fig 2). Assume *k* is not parallel to *l*, and denote the intersection points of the lines as follows: *A* ≔ *k* ∩ *d*, *B* ≔ *l* ∩ *d*, and *C* ≔ *k* ∩ *l*.

Let *c* denote the centroid of *D*, and let *p*(*c*) denote its projection onto the diagonal line *d*. In order to choose an “optimal” triangle *ABC*, we selecting the lines *k* and *l*, described above, in such a way that we minimize the sum of the following quantities: (1) the sum of squares of the distances of the points in *D* from the two lines, *k* and *l*, but we only consider 75% of the points each time: all the outer points and those inner points that are closest to the line; see the “10–75% zone” indicated in Fig 2 for the line *k*, (2) squared distance between *A* and *p*(*c*), and squared distance between *B* and *p*(*c*), and (3) squared height *h* of the triangle *ABC*; see the height *h* shown in Fig 2.

In the actual computations, we consider 50 equidistant candidates for the angle *φ* and 50 equidistant candidates for the angle *ψ*, and we make the lines *k* and *l* run through the points located at the 10th percentile in the direction perpendicular to the respective lines.

The topological triangle is a convenient visualization tool; however, for the purpose of statistical analysis, we need specific numbers, not a geometric figure. Therefore, we propose the following five descriptors of the topological triangle: (1) the length of the base *AB* of the triangle (*triangle width*) (2) the height *h* of the triangle (*triangle height*), (3) the location of the triangle represented by the *X* coordinate of the middle of the base of the triangle (*triangle location*), (4) the quotient of the lengths of the edges of the triangle: |*AC*|/|*BC*| (*triangle proportion*), and (5) the misalignment of the triangle equal the sum that is minimized while choosing the optimal angles *φ* and *ψ*, described above (*triangle misalignment*). The intuition behind these features of the persistence diagrams is described in details in Section 4.

In the classical topological data analysis, points located far away from the diagonal (which correspond to longer persistence intervals) are perceived as more significant, while points closer to the diagonal are considered noise. However, in the hrv analysis, the points that are near the diagonal may also play an important role, which we discuss in Section 4. Because of that, we let also these points contribute to the topological triangle that we construct.

### 2.3 Data sets and the method of processing and analyzing the data

ECG records of two groups of patients were analyzed:

- CON—a group consisting of 46 healthy individuals (28 men), aged 51 ± 12 years, (the data are available from [44–46]).
- STR—a group consisting of 41 patients (30 men), aged 55 ± 9 years, hospitalized due to the episode of acute ischemic stroke (the data are available from [47–49]).

The study complies with the Declaration of Helsinki; the study protocol was approved by the Ethics Committee of the Medical University of Gdańsk (NKEBN/422/2011 and NKEBN/190/2011). All the participants were informed about the study merits and signed a written consent.

The groups did not significantly differ according to age and sex. For each subject, ECG was recorded during rest in the supine position, and sequences of 512 intervals between successive heartbeats (RR intervals) were used for the analyses. All recordings were carefully inspected and edited by the cardiologist and RR series with the number of artifacts or ectopic beats exceeding 10% of all RR intervals were not included in the study. The ectopic beats constituted 0.2% of all the data and were present in the recordings of 11 individuals only.

**Fig 3:**
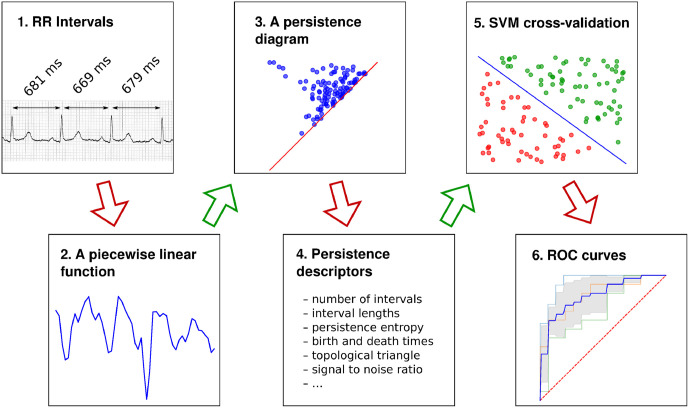
The steps 1–6 of data analysis described in Section 2.3.

The topological data analysis that we conducted can be summarized in the following six steps that are schematically depicted in Fig 3. It is a special case of a generic approach to data modeling based on persistent homology and machine learning, see e.g. [36, Fig 1].

1. We extract sequences of RR intervals (see Fig 3, part 1) from the ECG records in which arrhythmia artefacts were previously marked based on visual inspection of the ECG graphs. There is no universal method for editing the RR intervals; cf. [50]. Therefore, taking into account the specificity of persistent homology, we apply the following procedure. For the purpose of computing classical frequency-domain parameters, we use linear interpolation to smoothen the RRs that were not classified as normal, whenever possible. For the purpose of computing all the other indices, we only use normal RRs. Additionally, since it is known that topological methods are vulnerable to outliers (see e.g. [51]), we remove those RRs that are apart from the 25-75 percentile range by more than a quarter of the median of the RRs in the sample, provided their number does not exceed 4. If there are more than 4 outliers, all of them are considered part of the pattern and participate in the data analysis.
2. For each subject, the RR tachogram in terms of a piecewise linear function is obtained by joining consecutive values of RRs by affine segments. We remark that if we use a smooth function instead, such as the one obtained by cubic spline interpolation, the persistence of the sublevel sets would be essentially the same, so our choice of the simplest interpolation method does not cause any loss of generality.
3. We compute the persistence diagram for each function using the Dionysus 2 software [32, 33] applied to a filtration created from the RR points and segments in the function graph.
4. We compute topological indices for each persistence diagram, as defined in Section 2.2.
5. We construct machine learning models to assess the usefulness of the topological indices in relation to the most often used standard hrv parameters; see [19]. We consider the following four classical frequency domain parameters:
  - *total power*—the variance of RR intervals over the considered period [ms^2^],
  - *lfnu*—relative power of the low-frequency band (0.04–0.15 Hz) in normal units,
  - *hfnu*—relative power of the high-frequency band (0.15–0.4 Hz) in normal units,
  - *lf/hf ratio*—the ratio of the low-frequency power to the high-frequency power [ms^2^].
  We also compute the following four classical time domain parameters:
  - *mean nni*—mean value of normal-to-normal RR interval,
  - *sdnn*—standard deviation of normal-to-normal RR intervals,
  - *pnni 50*—percentage of successive RR intervals that differ by more than 50 ms,
  - *rmssd*—root mean square of successive RR interval differences.
  Specifically, we compare models built on the above quadruples of parameters with some of the best performing models out of the 420 tested models based on all the possible quadruples of the following 7 parameters extracted from persistence diagrams: the total number of persistence intervals (see Section 2.2), *length median*, *length stdev*, *length sum* (see Section 2.2.1), *frac5%* (see Section 2.2.4), *signal to noise* (see Section 2.2.5), and *pers entropy* (see Section 2.2.3), combined with up to 2 out of the 5 parameters derived from the topological triangle (see Section 2.2.8). We use the Support Vector Machine (SVM) classifier (see [52], §9.3) with the linear kernel. We conduct a 3-fold cross validation (see [52], §8.5.3) to compute receiver operating characteristic (ROC) curves (see [52], §8.3.6). In particular, we divide the set of the patients into three equal parts with a pseudo-random stratified k-folds cross-validator, then we use every set of two parts to train the model, and we use the third part for the evaluation and ROC curve computation. When cross-validating, each time we standardize the features by removing the mean and scaling to unit variance on training data, and then applying the same transformation to the test data. We use an implementation of the cross-validator, the standard scaler, and the SVM available in the scikit-learn library in Python [53]. We remark that a 5-fold cross validation is commonly used in the literature, but we prefer to use the 3-fold cross validation due to the relatively small sample size.
6. We average the three computed ROC curves to obtain the mean ROC curve, and then we take the area under the curve (AUC) as a single number that quantifies the accuracy of the model.

## 3 Results

**Fig 4:**
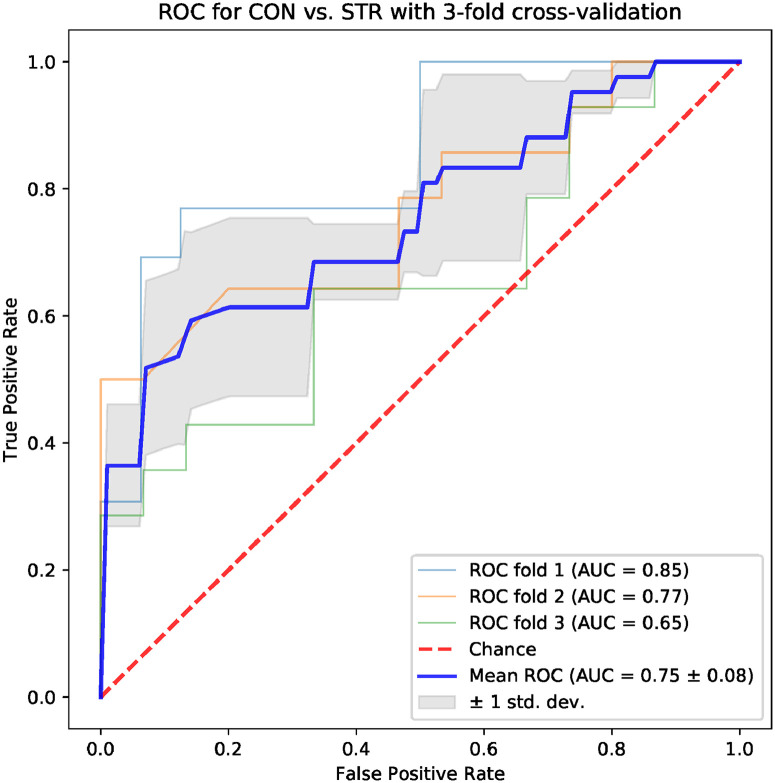
Results for the model trained with four frequency hrv parameters: *Lf/hf ratio*, *lfnu*, *hfnu*, *total power*.

**Fig 5:**
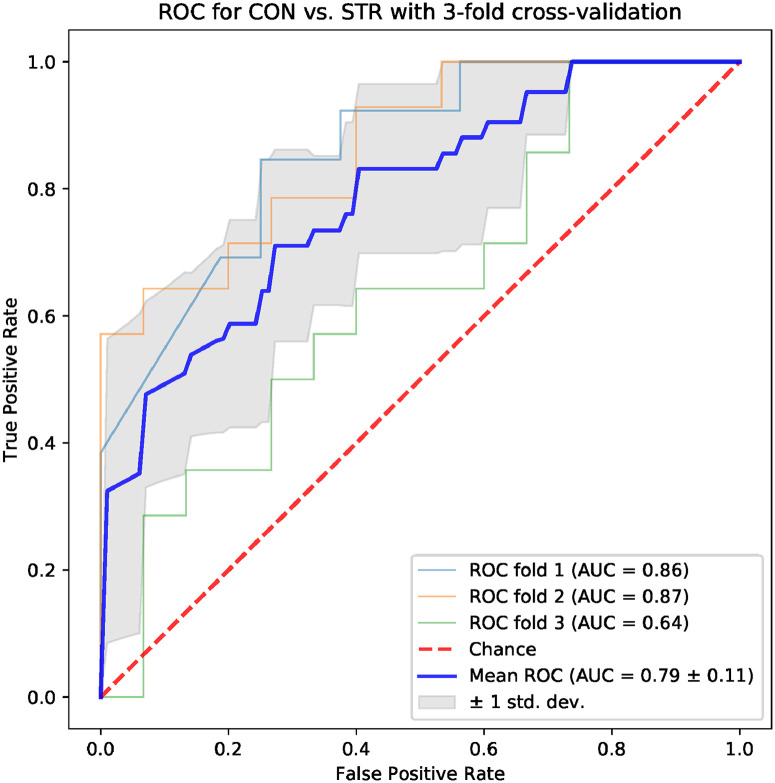
Results for the model trained with four classical hrv time domain parameters: *Mean nni*, *sdnn*, *pnni 50*, *rmssd*.

**Fig 6:**
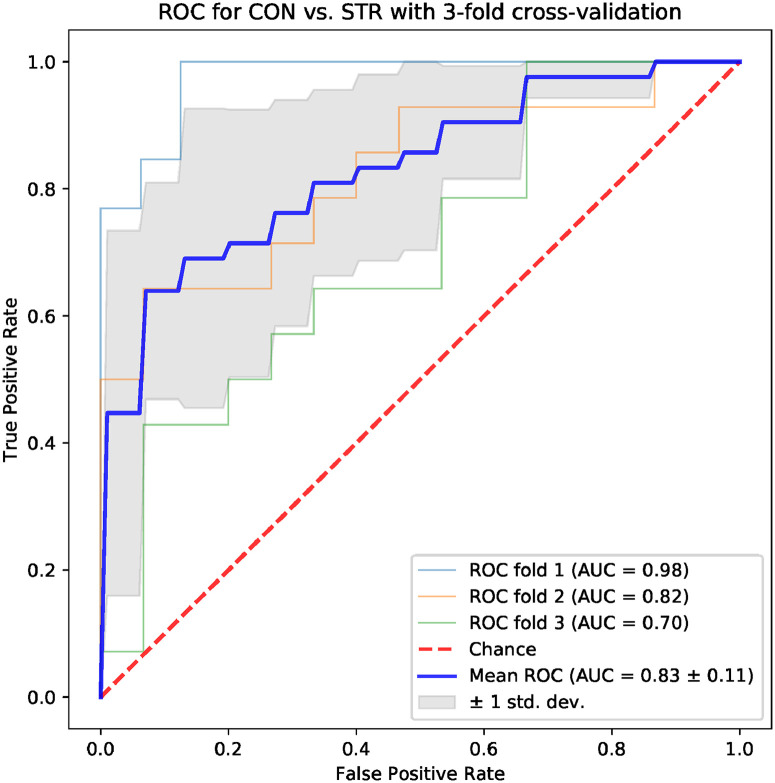
Results for the model trained with four topological parameters: *Triangle height, triangle location, number of intervals, length sum*.

**Fig 7:**
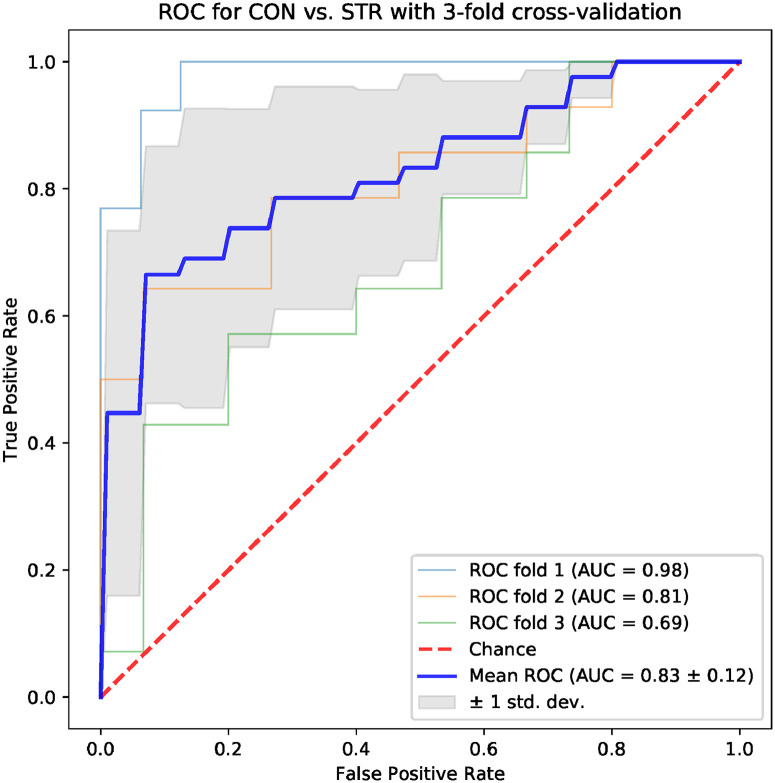
Results for the model trained with four topological parameters: *Triangle location, triangle misalignment, number of intervals, frac 5%*.

**Fig 8:**
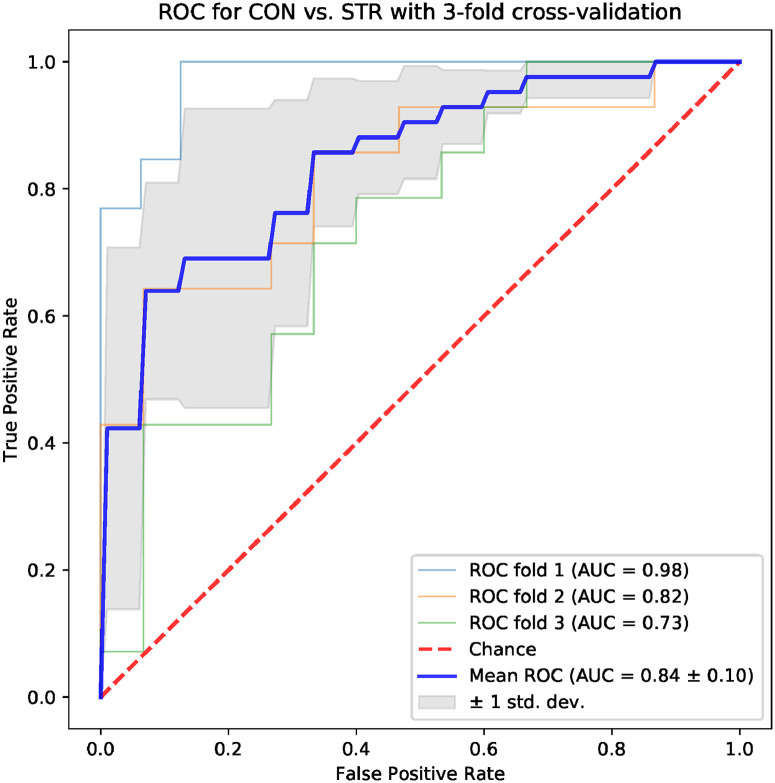
Results for the model trained with four topological parameters: *Triangle location, number of intervals, length sum, signal to noise*.

**Fig 9:**
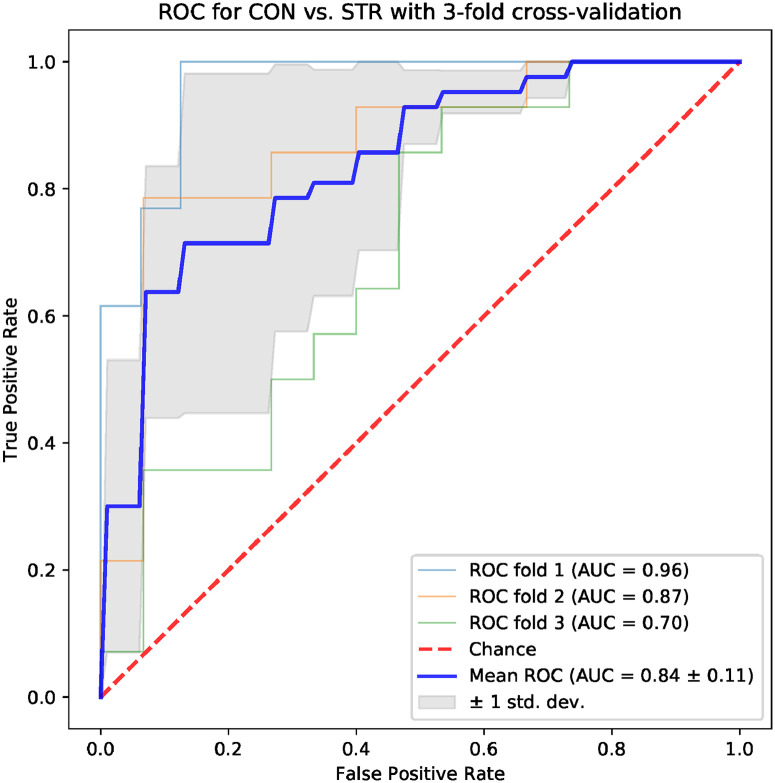
Results for the model trained with four topological parameters: *Triangle width, number of intervals, length sum, frac 5%*.

Machine learning models based on standard time-domain, frequency-domain and topological indices have been compared for their ability to differentiate the CON and STR groups. The results for models based on standard hrv parameters are shown in Figs 4 and 5: the area under the ROC curve (AUC) was 0.75 ± 0.08 for the frequency-domain parameters, and 0.79 ± 0.11 for the time-domain parameters. Examples of best models based on topological indices are given in Figs 6–9; in these cases the mean AUC was about 0.83–0.84. In general, the higher the ROC curve is located above the diagonal, the better, because this indicates a higher true positive rate in comparison to the false positive rate. The overall performance of the classifier is thus measured by the AUC. A classifier with AUC close to 0.5 would be essentially as good as random choice. In our case all the SVMs were better than that, and even some yielded the AUC in some of the tests within the cross-validation procedure close to the optimal value of 1. The results obtained for the SVMs trained on the selected topological features were somewhat better than in the case of the standard hrv parameters; the difference in the average AUC was between 5% and 12% in favor of the topological approach.

Out of the several topological indices, we chose a few that represented distinct features of persistence diagrams. The relation of the selected topological indices to standard heart rate variability parameters was tested in the CON group consisting of 46 healthy individuals by means of linear correlations. The results of this comparison are shown in Table 1.

The table shows the relationship between the topological indices and the most commonly used standard hrv parameters, both time-domain (*mean nni*, *sdnn*, *pnni 50* and *rmssd*) and frequency-domain (*lfnu*, *hfnu*, *lf/hf ratio* and *total power*) in healthy individuals (the CON group). One can notice strong positive linear correlation of time-domain hrv parameters (*sdnn*, *pnni 50* and *rmssd*) with topological indices based on the length of persistence intervals (*length median*, *length stdev* and *length sum*) as well as with *frac5%*, *triangle height*, *triangle misalignment* and *signal to noise*. *Triangle location* is strongly related with *mean nni* (correlation coefficient *R* = 0.98), while other considered topological indices show moderate (*number of intervals*, *length median*, *length stdev*, *length sum*, *triangle height*, *triangle misalignment*) or no correlation (*frac5%*, *triangle width*, *signal to noise*) with *mean nni*. In frequency-domain analysis, correlations of *total power* are similar as those for *sdnn*, which has a simple mathematical justification [19]. Other spectral parameters (*lfnu*, *hfnu*, *lf/hf ratio*) showed no correlation with *length stdev*, *signal to noise* and all the indices characterizing the topological triangle. The parameter characterizing vagal modulation (*hfnu*) showed moderate positive correlation with the following indices: *number of intervals*, *length median*, *length sum* and *frac 5%*. On the other hand, *lfnu* showed moderate negative correlation with these topological indices.

**Fig 10:**
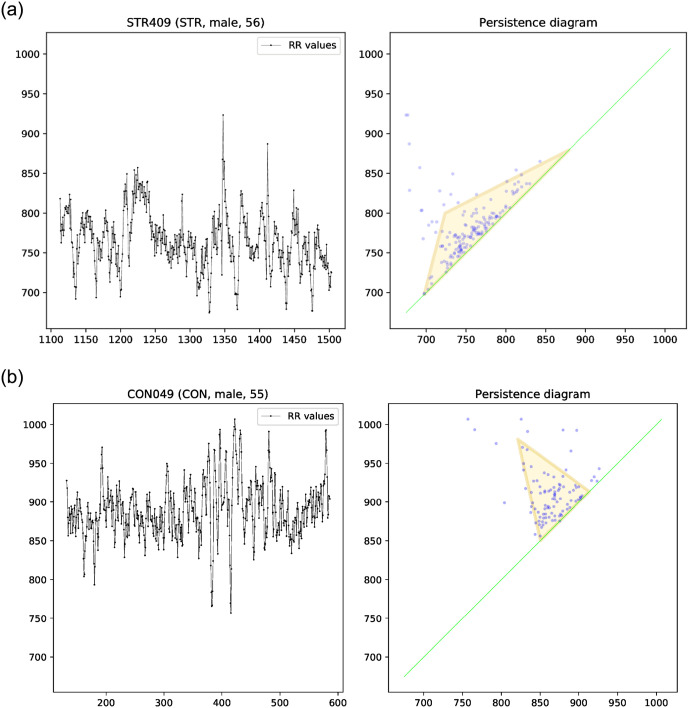
Comparison of tachograms (left) and persistence diagrams (right) in a patient with an acute ischemic stroke (STR, male, 56 years old, upper panel) and a healthy person (CON, male, 55 years old, bottom panel). See text for details.

An example that shows the advantage of topological indices in detecting altered heart rate variability is given in Fig 10 and Table 2. One healthy person (man, 55 years old) was compared with a stroke patient (man, 56 years old). In this example, almost all standard hrv parameters appeared to be similar, while several topological indices were considerably different. Unfortunately, this does not seem to be a general trend, because otherwise the ROC curves shown in Figs 6–9 would have been considerably better than those in Figs 4 and 5, while the difference is in fact not that profound. Nevertheless, this case study clearly highlights the fact that the topological features have a different potential for differentiating between the patients than the standard hrv parameters, and in some cases might be more appropriate.

**Table 2:** Comparison of values of all the considered standard and topological hrv parameters in a patient with the acute ischemic stroke (STR, male, 56 years, left-hand side) and a healthy person (CON, male, 55 years, right-hand side). Ten features that exhibit the highest relative differences are shown in bold typeface. See text for details.

(a) Frequency domain features	
lf	503.865 − 635.472
**hf**	**89.084 − 158.911**
lf hf ratio	5.656 − 3.999
lfnu	84.976 − 79.996
hfnu	15.024 − 20.004
total power	897.757 − 1018.908
vlf	304.808 − 224.525
(b) Time domain features	
mean nni	763.641 − 889.126
sdnn	36.428 − 36.801
sdsd	22.343 − 22.348
pnni 50	2.539 − 2.930
pnni 20	32.227 − 36.133
rmssd	22.344 − 22.348
median nni	761.758 − 886.675
range nni	248.646 − 249.760
cvsd	0.029 − 0.025
cvnni	0.048 − 0.041
mean hr	78.748 − 67.597
max hr	88.922 − 79.259
min hr	64.978 − 59.597
std hr	3.726 − 2.796
(c) Topological features	
number of intervals	154 − 97
longest interval	248.646 − 249.760
2/1 ratio	0.991 − 0.911
3/1 ratio	0.836 − 0.729
**length mean**	**29.653 − 47.873**
**length median**	**16.495 − 34.080**
length stdev	40.159 − 48.174
length sum	8.919 − 9.070
pers entropy	6.461 − 6.010
normed entropy	0.531 − 0.493
length threshold	46.170 − 50.339
**frac5%**	**0.136 − 0.330**
**frac100**	**0.058 − 0.113**
frac200	0.019 − 0.021
middle mean	110.236 − 99.307
middle stdev	60.120 − 51.961
**signal to noise**	**1.028 − 2.168**
birth mean	711.598 − 843.328
birth stdev	25.522 − 31.586
death mean	821.834 − 942.635
death stdev	44.310 − 33.574
**triangle width**	**257.161 − 89.728**
**triangle height**	**53.928 − 113.149**
triangle location	788.270 − 881.985
**triangle proportion**	**0.607 − 1.170**
**triangle misalignment**	**311.368 − 122.318**

## 4 Discussion

We are interested in a new approach to the analysis of heart rate variability. This approach should not be restricted to interpreting beat-to-beat variations, but should provide an insight into changes that appear in sequences of RR intervals. Moreover, it should not be limited to the analysis of chunks of predefined length; note that such a restriction appears in some methods, for example, in permutation pattern analysis [54]. Topological indices based on persistence diagrams seem to respond to these needs. As a result, with these methods we can evaluate changes in heart rhythm caused by different mechanisms.

The persistent homology method has some similarities with the analysis of the statistics of so-called *U-shape patterns* (see [55, 56] and references therein). U-shapes are parts of the tachogram in the night-time recordings consisting of relatively short periods of smooth accelerations followed by decelerations in the heart rhythm, lasting in total approximately 20–40 seconds. So-called *short acceleration-deceleration events* [56] show some similarities with U-shape patterns but could not be classified as such due to their shorter duration, symmetric acceleration, or smaller relative amplitude. Persistent homology methods provide a more general approach in which each U-shape or short acceleration-deceleration event is related to a persisting connected component of the graph built from the RR data (a persistence interval). However, as a multiscale approach, persistent homology detects all the appearing U-shapes irrespective of their duration and amplitude, while classifying them by the amplitude and ignoring the information on their duration.

There is a great variety of topological indices used in the analysis of time series data; see e.g. [36]. We have chosen a set of topological indices that seem to be useful for hrv analysis. Many of these indices provide information similar to time-domain standard hrv parameters and *total power* in the frequency analysis (cf. Table 1). These indices especially include those based on the length of persistence intervals (*length median*, *length stdev* and *length sum*); in these cases, the value of the index grows with the increase in heart rate variability. At the same time, these indices exhibit weak or moderate linear correlation with the mean length of the RR intervals. However, if one considers the relation of the mentioned indices to spectral components of hrv then one encounters a different pattern. While *length stdev* is not related to them, *length median* and *length sum* show moderate positive linear correlation with *hfnu*, and moderate negative linear correlation with *lfnu* and *lf/hf*. Taking all this into consideration, we are inclined to believe that indices based on the length of persistence intervals mostly represent the parasympathetic influence on the heart rate.

We have introduced new parameters derived from the persistence diagrams. We first noticed that the points in the persistence diagram in almost all the cases formed the shape of a triangle of various height, width, location, and slantingness. We made these features into numeric parameters and tested whether they had some relation to the standard hrv features. We hope that these new parameters, especially in combinations, might deliver comprehensive information about heart rate changes.

The intuition behind persistence diagrams implies that the location of the triangle closely follows the average RR value; indeed, there is a strong correlation between these parameters (R = 0.98; see Table 1). The width of the triangle reflects the width of the range of RR values and is correlated with *sdnn* (R = 0.59). On the other hand, the height of the triangle measures the quantity of long persistence intervals, and thus corresponds to large variations in RR values spread over multiple RRs. In standard hrv analysis the information about large changes of the consecutive RR values is mostly given by *pnni 50* and indeed, there is a strong correlation between the triangle height and *pnni 50* values (R = 0.86). If the triangle is slanted rightwards then this indicates the prevalence of larger oscillations within high range of RR values, while the triangle slanted leftwards suggests that there are more oscillations within the low range of RRs. Eventually, the triangle misalignment parameter measures the discrepancy between the actual shape of the set of points in the persistence diagram and the shape of the approximating triangle.

Another parameter introduced in the paper is the *signal to noise* ratio. Its definition is inspired by the notion of signal-to-noise ratio in science and engineering, where it measures the proportion between the level of desired signal and the level of background noise. Typically, when considering topological persistence in general, small fluctuations in data are regarded as noise, and thus one would only focus on long persistence intervals. However, in the analysis of hrv, small persistence intervals may also carry important information, reflecting very frequent oscillations of zig-zag type with small amplitude, in particular 2UV class of patterns according to Porta et al. classification [57]. Therefore, low level of signal-to-noise ratio in our case might be an indication of the dominance of this kind of short-term oscillations over long-term changes in RR values.

Out of the other parameters that appeared important in studying RR series, we would like to emphasize the number of persistence intervals. It seems to mirror some special features of heart rate variability that are encountered especially in stroke patients. In the STR group, the number of persistence intervals was significantly higher than in the CON group and had no relation to any of the standard hrv parameters. This might mean that the number of persistence intervals reflects heart rate changes mediated not only by autonomic nervous system but also by other mechanisms (respiratory, humoral, etc.)

One of the aims of the paper is to evaluate the usefulness of the topological indices in practical hrv analysis. For that end, we conducted an experiment in which we tested multiple models based on 4 parameters for their ability to differentiate CON and STR groups using a representative machine learning technique, namely the SVM. The results that we obtained were encouraging. In the tests conducted with the sets of the standard time-domain and frequency-domain hrv parameters resulted in the AUC mean values not exceeding 0.79 (see Figs 4 and 5). However, many sets of topological indices yielded mean AUC values of 0.83 and more (see some examples in Figs 6–9). There are a few indices that repeatedly appear in the best predictive models: *number of intervals*, *length median*, *length stdev*, *length sum*, *triangle height*, *triangle width*, *triangle location*, *frac5%*, and *signal to noise*. Surprisingly, *pers entropy*, which is a well-established measure of complexity, is not included in this list. Persistent entropy is known to be a very good topological classifier in some other applications, for example, in epilepsy detection [35]. However, in our case the considered variant of entropy was not so useful. Further investigation of this finding is needed.

In order to get some insight into the usefulness of the topological indices, we show a specific case (see Table 2 and Fig 10) in which the indices based on persistence diagrams turn out to be a better tool for the differentiation between RR series than the standard hrv parameters. This example suggests that the topological indices provide another kind of information, especially in groups that encounter hrv changes caused by various mechanisms.

Finally, let us point out possible further research perspectives in the discussed topic. The persistent homology techniques may be applied to studying some pathological states that are difficult to analyze by other tools. In particular, in our preliminary study [27], we applied a simplified version of the currently introduced method to the problem of characterizing a group of patients suffering from an episode of transient ischemic attack, with promising results; however, extended study on a larger group of subjects is needed. Another interesting direction of research is to confront the topological methods with other approaches. In our paper we did it for standard hrv measures, but it would be valuable to study the relation of persistent homology approach with methods based on non-linear analysis, chaos theory and dynamical systems, in particular to compare the topological indices with such quantities as DFA, Lyapunov Exponent, Correlation Dimension, various kinds of entropy measures, to mention some examples.

Let us remark that we studied the case of 0-dimensional homology, i.e., we examined connected components of the graph of a map constructed from RR series. A natural next step of research is to consider higher-dimensional counterparts of this construction that include the analysis of persistence diagrams in dimensions 2, 3, and above. In this approach we would study the geometry of data in higher dimensions that might reveal additional information about its structure. Moreover, it seems that involving more sophisticated methods of Topological Data Analysis, such as Persistence Landscapes [58], or Persistence Images [59], might provide new insights, and might result in the development of more powerful approaches to hrv analysis.

## 5 Conclusion

Topological indices based on the analysis of persistence diagrams are able to assess heart rate variability and can be useful in the detection of pathological states. It seems that topological indices might capture more features of heart rate changes than standard hrv parameters and might provide valuable information about mechanisms involved in cardiovascular regulation. Additional studies are needed to further validate these findings.
